# Neuroretinal Rim Area and Body Mass Index

**DOI:** 10.1371/journal.pone.0030104

**Published:** 2012-01-09

**Authors:** Liang Xu, Ya X. Wang, Shuang Wang, Jost B. Jonas

**Affiliations:** 1 Beijing Institute of Ophthalmology, Beijing Tongren Hospital, Capital University of Medical Science, Beijing, China; 2 Department of Ophthalmology, Faculty of Clinical Medicine Mannheim, University of Heidelberg, Mannheim, Germany; Institute Biomedical Research August Pi Sunyer (IDIBAPS) - Hospital Clinic of Barcelona, Spain

## Abstract

**Purpose:**

To examine associations between neuroretinal rim area, pressure related factors and anthropometric parameters in a population-based setting.

**Methods:**

The population-based cross-sectional Beijing Eye Study 2006 included 3251 subjects with an age of 45+ years. The participants underwent a detailed ophthalmic examination. Exclusion criteria for our study were high myopia of more than -8 diopters and angle-closure glaucoma.

**Results:**

The study included 2917 subjects with a mean age of 59.8±9.8 years (range: 45–89 years). Mean neuroretinal rim area was 1.97±0.38 mm^2^, mean intraocular pressure 15.6±3.0 mmHg, mean diastolic blood pressure 79.0±5.9 mm Hg, mean systolic blood pressure 133.5±11.1 mmHg, and mean body mass index was 25.5±3.7. In univariate analysis, neuroretinal rim area was significantly associated with optic disc size, open-angle glaucoma, refractive error, age and gender. After adjustment for these parameters in a multivariate analysis, a larger neuroretinal rim area was significantly correlated with a higher body mass index (*P*<0.001), in addition to be associated with a lower intraocular pressure (*P* = 0.004), lower mean blood pressure (*P* = 0.02), and higher ocular perfusion pressure.

**Conclusions:**

In a general population, neuroretinal rim as equivalent of the optic nerve fibers is related to a higher body mass index, after adjustment for disc area, refractive error, age, gender, open-angle glaucoma, intraocular pressure, blood pressure and ocular perfusion pressure. Since body mass index is associated with cerebrospinal fluid pressure, the latter may be associated with neuroretinal rim area. It may serve as an indirect hint for an association between cerebrospinal fluid pressure and glaucoma.

## Introduction

The neuroretinal rim is the intrapapillary equivalent of the optic nerve fibers. It is one of the most important morphologic parameters to detect glaucomatous optic neuropathy and to grade the amount of glaucomatous optic nerve damage [1]. Numerous hospital-based studies have shown the association between a loss of neuroretinal rim and the height of intraocular pressure in patients with glaucoma [2]–[5]. The population-based Baltimore Eye Survey and the Early Manifest Glaucoma Trial revealed correlations between a smaller size of the neuroretinal rim and a higher level of intraocular pressure in large non-hospital based populations [6], [7]. Other studies have suggested that pressure parameters others than intraocular pressure also have an influence on the neuroretinal rim size. These pressure parameters included the arterial blood pressure and the ocular perfusion pressure as a combination of blood pressure and intraocular pressure [8]–[13]. Recent studies have added a new parameter, the cerebrospinal fluid pressure, to the list of pressure related variables with a potential influence on the optic nerve and the development of glaucomatous optic neuropathy [14]–[20]. In view of these new findings, we conducted our study to examine in the unselected large sample of a population-based investigation whether the neuroretinal rim, besides to be correlated with intraocular pressure, is additionally associated with the other pressure parameters mentioned above. Since a direct measurement of the cerebrospinal fluid pressure is invasive and since the cerebrospinal fluid pressure is associated with the body mass index [21], [22], we took the body mass index as surrogate for the cerebrospinal fluid pressure. The results of the study could be helpful to further elucidate whether and to which extent the pressure related parameters are associated with the neuroretinal rim, and due to the association between neuroretinal rim area and glaucoma, to indirectly elucidate whether the pressure related parameters are associated with glaucomatous optic neuropathy.

## Methods

### Ethics Statement

The Medical Ethics Committee of the Beijing Tongren Hospital approved the study protocol, and all participants provided informed consent.

The population-based Beijing Eye Study 2006 was the follow-up study of the Beijing Eye Study 2001. In brief, at baseline in 2001, 4439 subjects of 5324 eligible individuals were included. At the follow-up study in 2006, 3251 subjects (1838 women) participated, corresponding to a response rate of 73.2%. The rural part of the study in 2006 consisted of 1500 (46.1%) subjects and the urban part of 1751 (53.9%) subjects. Mean age was 60.4±10.0 years (range, 45 – 89 years). The study design has been described in detail previously [23], [24]. In the study of 2006, a comprehensive eye examination was carried out, including visual acuity assessment, frequency doubling perimetry (screening program C-20-1; Zeiss-Humphrey, Dublin, CA), noncontact tonometry (CT-60 computed tonometer, Topcon Ltd., Tokyo, Japan), slit-lamp examination of the external eye and anterior segment, and photography of the lens (Neitz CT-R camera, Neitz Instruments Co., Tokyo, Japan), the macula and the optic disc (CR6-45NM, Canon Inc. Tokyo, Japan). All examinations were carried out in the communities, either in schoolhouses or in community houses. The blood pressure was measured with the participant sitting for at least 5 min. The study participants had refrained from smoking and drinking of coffee, tea, or alcohol for at least 3 h. In addition, any exercise was not performed for the last 30 min prior to the blood pressure measurements. A standardized mercury sphygmomanometer was used, and the cuff size was chosen according to the measured circumference of the upper arm. Arterial hypertension was defined as a systolic blood pressure ≥140 mm Hg and/or a diastolic blood pressure ≥90 mm Hg, and/or self-reported current treatment for arterial hypertension with antihypertensive medication. The self-reported therapy of arterial hypertension was assessed in a questionnaire which included questions on the socioeconomic and medical background. Mean arterial blood pressure was defined as “Diastolic Blood Pressure + 1/3×(Systolic Blood Pressure – Diastolic Blood Pressure)”. Ocular perfusion pressure was calculated as 2/3× (Mean Arterial Blood Pressure – Intraocular Pressure). The body height was determined in a standardized manner with the shoes routinely removed. The subjects were asked to stand upright as much as possible and with the head raised upright as much as possible. We used a stadiometer as measuring instrument. The floor was completely even. We did not take into account nor corrected age-related reductions in height of subjects who reportedly were taller during their middle-age. Additionally, we determined the body weight and calculated the body mass index as ratio of body weight (measured in kilogram) divided by the square of the body height (measured in meters).

The optic disc photographs were digitalized. The digitized optic disc photographs were measured by outlining the optic disc border on the computer screen and using a planimetric software program. As described recently [24], the magnification by the optic media of the eye were corrected according to Littmanńs method taking into account the refractive error. In a second step of the examination, the width of the neuroretinal rim and the diameters of the optic cup and optic disc were measured in the vertical meridian [25]. The vertical cup / disc diameter ratio and the optic cup area were calculated. The neuroretinal rim area resulted as difference of disc area minus cup area.

Glaucoma was defined according to the criteria of the International Society of Geographic and Epidemiological Ophthalmology ISGEO [26]. As described in detail previously [25], the whole glaucoma group was then differentiated by gonioscopy or anterior segment optical coherence tomography into subjects with open-angle glaucoma or subjects with angle-closure glaucoma.

Inclusion criteria for the present study were the availability of optic disc photographs and measurements of intraocular pressure, blood pressure and body mass index. We excluded patients with angle-closure glaucoma to avoid a potentially confounding effect of the marked intraocular pressure elevation by the blockage of the anterior chamber angle due to primarily intraocular causes. We also excluded all highly myopic eyes (defined as a myopic refractive error of more than -8 diopters), since high myopia is associated with a stretching of the posterior pole and a secondary enlargement of the optic disc and neuroretinal rim [27].

Statistical analysis was performed using SPSS (IBM-SPSS for Windows, version 19.0, SPSS, Chicago, IL). Only one randomly selected eye per subject was taken for the statistical analysis. Continuous data were presented as mean±standard deviation. Linear regression models were used to investigate the associations of the size of the neuroretinal rim with the continuous (e.g., intraocular pressure) or categorical outcomes (e.g., gender). The 95% confidence intervals (CI) are described. All *P*-values were 2-sided and were considered statistically significant when the values were less than 0.05.

## Results

The study included 2917 (89.7%) subjects. The mean age was 59.8±9.8 years (range: 45 – 89 years), and the mean refractive error was −0.07±1.60 diopters (range: −8.00 diopters to +7.25 diopters). Due to the exclusion criteria, the study participants as compared to the subjects not included into the study were significantly (*P*<0.001) less myopic (−0.07±1.60 diopters versus −2.26±5.25 diopters), and they were significantly (*P*<0.001) younger (59.8±9.8 years versus 65.4±11.1 years).

Mean neuroretinal rim area was 1.97±0.38 mm^2^, mean intraocular pressure 15.6±3.0 mmHg, mean diastolic blood pressure 79.0±5.9 mm Hg, mean systolic blood pressure 133.5±11.1 mmHg, and the mean body mass index was 25.5±3.7 kg/m^2^.

In univariate analysis, a larger neuroretinal rim area was significantly associated with higher age, female gender, absence of open-angle glaucoma, larger optic disc size and more hyperopic refractive error, higher body mass index, lower intraocular pressure, and lower systolic, diastolic and mean blood pressure (Table 1). It was not significantly associated with body height and weight.

**Table 1:** Associations between Pressure Related Parameters, Anthropometric Parameters and Neuroretinal rim Area in the Beijing Eye Study 2006 (Univariate Analysis).

Parameter	*P*-Value	Standardized Corre-lation Coefficient Beta	Coefficient Regression	95% Confidence Interval of Regression Coefficient
Age (Years)	0.001	0.06	0.002	0.001, 0.004
Gender	0.02			
Presence of Open-Angle Glaucoma	<0.001			
Optic Disc Area (mm^2^)	<0.001	0.63	0.48	0.46, 0.50
Refractive Error (Dpt)	<0.001	0.08	0.02	0.01, 0.03
Body Mass Index (kg/m^2^)	0.04	0.04	0.004	0.001, 0.008
Body Height (cm)	0.64			
Body Weight (kg)	0.10			
Intraocular Pressure (mmHg)	<0.001	-0.10	-0.013	-0.017, -0.008
Systolic Blood Pressure (mmHg)	0.004	-0.05	-0.002	-0.003, -0.001
Diastolic Blood Pressure (mmHg)	<0.001	-0.08	-0.005	-0.008, -0.003
Mean Arterial Blood Pressure (mmHg)	<0.001	-0.08	-0.004	-0.006, -0.002
Ocular Perfusion Pressure (mmHg)	0.13	-0.03	-0.001	-0.002, 0.000

We then performed a multivariate analysis which included the neuroretinal rim area as dependent variable and all parameters as independent variables for which the *P*-value was <0.15 in the univariate analysis. In a first step, we adjusted the neuroretinal rim area for optic disc size and refractive error, since both parameters were associated with neuroretinal rim due to the definition and calculation of rim area. We then adjusted for gender and age, since both systemic parameters were associated with neuroretinal rim area. Additionally we adjusted for the presence or absence of open-angle glaucoma, since glaucoma leads to a loss of neuroretinal rim. In a second move, we added in a stepwise manner intraocular pressure, mean blood pressure and ocular perfusion pressure, and then body mass index. It showed that, after the adjustment for optic disc area, refractive error, age, gender and presence of open-angle glaucoma, neuroretinal rim area was significantly associated with higher body mass index (*P*<0.001), in addition to lower intraocular pressure (*P* = 0.004), lower mean arterial blood pressure (*P* = 0.02), and marginally significantly, with higher ocular perfusion pressure (*P* = 0.068) (Table 2).

**Table 2:** Multivariate Analysis of the Associations between Pressure Related Parameters, Anthropometric Parameters and Neuroretinal rim Area in the Beijing Eye Study 2006, Adjusted for Optic Disc Size, Refractive Error, Presence of Glaucomatous Optic Neuropathy, and Gender.

Parameter	*P*-Value	Regression Coefficient	Standardized Coeff. Beta	95% Confid. Interval of Regr. Coeff	Collinearity Analysis Variance Inflation Factor
Body Mass Index (kg/m^2^)	<0.001	0.006	0.05	0.003, 0.009	1.14
Intraocular Pressure (mmHg)	0.004	-0.006	-0.05	-0.011, -0.002	1.44
Mean Arterial Blood Pressure (mmHg)	0.02	-0.004	-0.08	-0.008, -0.001	6.17
Ocular Perfusion Pressure (mmHg)	0.068	0.002	0.06	0.000, 0.005	6.19
Optic Disc Area (mm^2^)	<0.001	0.48	0.63	0.46, 0.50	1.03
Presence of Open-Angle Glaucoma	<0.001	-0.46	-0.18	-0.53, -0.39	1.01
Gender	0.04	0.02	0.03	0.001, 0.05	1.05
Age (Years)	0.42				
Refractive Error (Dpt)	0.57				

## Discussion

In this population-based study of adult Chinese, we found that with highly myopic subjects and patients with angle-closure glaucoma excluded, neuroretinal rim was significantly associated with higher body mass index. This association between larger neuroretinal rim area and higher body mass index persisted after adjustment for disc area, refractive error, age, gender, open-angle glaucoma, intraocular pressure, blood pressure and ocular perfusion pressure.

Considering a decreased neuroretinal rim area as surrogate for glaucomatous optic nerve damage, the results of our study agree with previous investigations in which a lower body mass index was associated with a higher prevalence and incidence of glaucoma [28]. Pasquale and colleagues assessed the relationship between anthropometric measures and incident primary open-angle glaucoma in 78,777 women of the Nurses' Health Study and in 41,352 men of the Health Professionals Follow-up Study. They found that among women, a higher body mass index was associated with a lower risk of primary open-angle glaucoma with intraocular pressure readings of 21 mmHg or less at diagnosis [29]. In the Barbados Eye Study, persons most likely to have open-angle glaucoma were older men and had a family history of open-angle glaucoma, high intraocular pressure, lean body mass, and cataract history [30]. In a similar manner, Zheng and colleagues in the Singapore Malay Eye Study found that persons who were taller or had lower body mass index had a smaller neuroretinal rim area and a larger optic cup-to-disc area ratio [31]. Other studies such as the investigation by Gasser and colleagues did not find clear associations between obesity and the prevalence of glaucoma [32]. The results of our study partially contradict a recent longitudinal cohort study by Newman-Casey and colleagues [33]. That study included more than 2 million beneficiaries with an age of ≥40 years, who were continuously enrolled in a managed care network and who had 1 or more visits to an eye care provider during the period of 2001 to 2007. Using billing codes to identify individuals with open-angle glaucoma, the authors found in a multivariable regression model, that obese women as compared with non-obese women had a 6% increased hazard of developing open-angle glaucoma (adjusted hazard ratio: 1.06 [95% confidence interval (CI): 1.02–1.10]), while obese men as compared with non-obese men had no significant increased hazard of developing open-angle glaucoma OAG (adjusted hazard ratio: 0.98 [95% CI, 0.94–1.03]).

The association between lower body mass index and smaller neuroretinal rim area (or as a corollary with a higher prevalence and incidence of glaucoma) may be explained by the association between body mass index and cerebrospinal fluid pressure [21], [22]. A high body mass index through an elevated cerebrospinal fluid pressure as trans-lamina cribrosa counter pressure may compensate for an elevated intraocular pressure and thus protect against a loss of neuroretinal rim or development of glaucomatous optic neuropathy. The result of our study as well as the findings of previous investigations on associations between low body mass index and higher prevalence of open-angle glaucoma support the theory that a low cerebrospinal fluid pressure may play a role in the pathogenesis of glaucomatous optic neuropathy [14]–[20].

The association between high body mass index and large neuroretinal rim area appears to be contradicted by reported associations between high body mass index and elevated intraocular pressure in arterial hypertensive patients, who, however, did not show an increased prevalence of open-angle glaucoma [34], [35]. The elevated intraocular pressure in these obese patients with arterial hypertension may be compensated for by a simultaneous elevation of the cerebrospinal fluid pressure due to the physiologic correlation between cerebrospinal fluid pressure and arterial blood pressure [18].

Potential limitations of our study should be mentioned. First, the cross-sectional design of our study prevented inferring causality or a chronological order of changes. The temporal relationship between neuroretinal rim area and body mass index remains, therefore, uncertain. Second, as in other population-based studies that have used the ISGEO scheme to diagnose glaucoma, a small proportion of the persons with pseudonormal optic cups (e.g., minicups) and normal intraocular pressure may have been misclassified as nonglaucomatous and could thus have influenced the results of the multivariate analysis. Third, it has not universally been accepted that body mass index is correlated with cerebrospinal fluid pressure and may thus be taken as a surrogate for the cerebrospinal fluid pressure. Fourth, although the association between an increased body mass index and neuroretinal rim area was found to be statistically significant after correcting for other factors, one has to consider that in view of the relatively low regression coefficient, the strength of the relationship was not strong. Fifth, the mean body mass index with 25.5±3.7 kg/m^2^ was relatively high and slightly beyond the limit of overweight (globally defined as a body mass index ≥25 kg/m^2^) [36]. It remains to be shown whether the association between neuroretinal rim area and body mass index as demonstrated in our study can also validated in populations with a relatively low body mass index. The strengths of our study include its large population-based design and measurement of optic disc parameters.

In summary, in our cohort of Chinese persons aged 45 to 89 years, a larger neuroretinal rim as the equivalent of a higher number of optic nerve fibers was related to higher body mass index, after adjustment for disc area, refractive error, age, gender, open-angle glaucoma, intraocular pressure, blood pressure and ocular perfusion pressure. Since body mass index is associated with cerebrospinal fluid pressure, the latter may be associated with neuroretinal rim area. It may serve as an indirect hint for the association of the cerebrospinal fluid pressure with glaucoma.
